# Pollution Characteristics and Health Risk Assessment of Heavy Metals in Agricultural Soils over the Past Five Years in Zhejiang, Southeast China

**DOI:** 10.3390/ijerph192214642

**Published:** 2022-11-08

**Authors:** Jie Xiang, Peiwei Xu, Weizhong Chen, Xiaofeng Wang, Zhijian Chen, Dandan Xu, Yuan Chen, Mingluan Xing, Ping Cheng, Lizhi Wu, Bing Zhu

**Affiliations:** 1 Department of Environmental Health, Zhejiang Provincial Center for Disease Control and Prevention, Hangzhou 310000, China; 2 Hangzhou Center for Disease Control and Prevention, Hangzhou 310000, China

**Keywords:** heavy metals, agricultural soils, spatiotemporal distribution, health risk assessment

## Abstract

Heavy metal contamination in agricultural soils has attracted increasing attention in recent years. In this study, 1999 agricultural soil samples were collected from 11 cities in Zhejiang Province from 2016 to 2020, and the spatial and temporal variation characteristics of 3 of the most important heavy metals, i.e., lead (Pb), cadmium (Cd), and chromium (Cr) were analyzed. The results showed that Cd had a slightly higher sample over-standard rate of 12.06%. Spatial distribution and temporal trends showed that the Pb concentrations overall increased from 2016 to 2020 and mainly accumulated in southern Zhejiang. In addition, multiple exposure routes were evaluated for human health risks. Children are more susceptible to the adverse effects of heavy metals in agricultural soils, and oral ingestion was the major exposure route. Cr poses higher human health risks to humans than Pb and Cd in agricultural soils. Therefore, more rigid environmental monitoring and related soil remediation counter-measures for some sites with high concentrations of heavy metals are necessary to limit the potential threat to human health.

## 1. Introduction

Over the past few decades, heavy metal pollution of agricultural and urban soils has become a global concern because of its harmful effects on the ecological environment, as well as human health. For example, the United States, Russia, Australia, France, Spain, and India are threatened by cadmium (Cd)-contaminated soils [1,2,3]. In addition, lead (Pb), chromium (Cr) and other heavy metals in soils in America, Africa, and other continents also pose health hazards to humans [4,5]. Similarly, the quality of agricultural soils in China is of particular concern. According to a 2014 national soil survey by the Ministry of Ecology and Environment and the Ministry of Land and Resources of the People’s Republic of China, 16.1% of the point sites exceeded the standard in the survey area of about 6.3 million km^2^ of soil. The over-standard rate of soil point positions in cultivated land has reached 19.4%, which is obviously higher than that of woodlands and grasslands [6]. In particular, the over-standard rates for eight inorganic pollutants, including Cd, Hg, As, Cu, Pb, Cr, Zn, and Ni were 7.0%, 1.6%, 2.7%, 2.1%, 1.5%, 1.1%, 0.9%, and 4.8%, respectively [6]. Recent surveys have found that over the past 20 years, China’s agricultural soils have been contaminated with a variety of heavy metals [2,7]. The presence of heavy metals in soils might influence microbial diversity, and the presence of some potential pathogens [8,9,10]. In addition, the accumulated heavy metals can be transferred to crops, plants and other parts of the food chain and living environment, which may ultimately pose threats to human health [11,12,13].

According to related reports, Cd is a ubiquitous environmental toxicant and is recognized as a Group I human carcinogen by the International Agency for Research on Cancer [14]. Cd may cause kidney, liver, and bone damage through the consumption of Cd-contaminated crops. Cr is also considered as one of the most adverse substances to the environment, and hexavalent Cr (Cr (VI)) is a potential human carcinogen for which there is sufficient evidence of adverse effects on human organs and tissues [15,16]. Similar to Cr, Pb is also considered a neurotoxin that can damage vital body systems, such as the blood, cardiovascular, and nervous systems, via seafood, vegetables, and rice [11,17,18]. Therefore, it is extremely important to investigate the current levels of heavy metal contamination in soils, especially in agricultural soils.

The accumulation of heavy metals in agricultural soils is mainly due to industrial emissions, fuel combustion, waste management and transport, fertilizer and pesticide use, as well as wastewater irrigation in farmland [19,20,21,22,23]. The Yangtze River Delta region (YRDR), including Zhejiang Province and its three adjacent provinces and municipalities (Jiangsu, Anhui, and Shanghai), is one of the most cultivated regions in the world, and is also one of the most dynamic areas of economic development in China. During the past few decades, the agricultural soils in the YRDR have been reported to be polluted by heavy metals [21,24]. As Zhejiang is a key economic area in the YRDR, the province also is at risk from the contamination of heavy metals in agricultural soils. According to previous reports, there are different levels of heavy metal contamination in agricultural soils around chemical industrial zones, electronic waste dismantling centers, mining areas, watersheds, and other special areas in Zhejiang Province [25,26,27,28]. However, most related studies have mainly focused on a typical area and specific time, and the investigation on the current circumstance and potential human health risk of typical heavy metal pollution in agricultural soils remains limited. Therefore, it is critical to evaluate the temporal and spatial variation in heavy metals concentration of agricultural soils in Zhejiang Province.

Therefore, the objectives of this study were (1) to measure the concentrations of heavy metals in agricultural soils in Zhejiang Province, (2) to examine the temporal and spatial variations in heavy metals in agricultural soils, and (3) to estimate the potential health risks associated with heavy metal-contaminated soils. This study focused on the current status and health risk assessment of three of the most important heavy metals in the agricultural soils in Zhejiang, and could provide more basic and powerful evidence for the enhanced environmental monitoring and soil remediation.

## 2. Materials and Methods

### 2.1. Study Area

**Figure 1:**
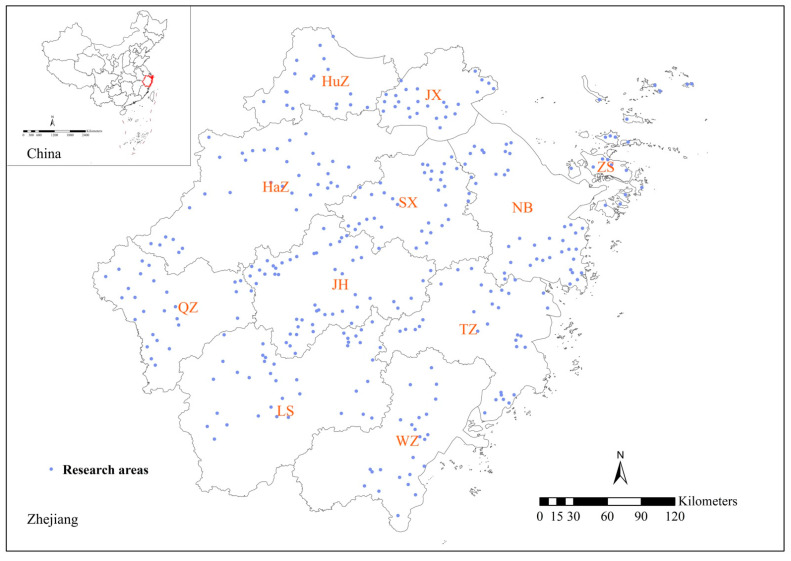
Study area and sampling located townships. (HaZ: Hangzhou, NB: Ningbo, WZ: Wenzhou, JX: Jiaxing, HuZ: Huzhou, SX: Shaoxing, JH: Jinhua, QZ: Quzhou, ZS: Zhoushan, TZ: Taizhou, LS: Lishui).

Zhejiang Province is situated in southeast China, south of the Yangtze River Delta (Figure 1). The province has a humid subtropical monsoon climate with an average annual rainfall of 1560.4 mm and an annual average temperature of 18.4 °C. Zhejiang Province has undulating terrain, which is covered by mountainous areas, hilly basins, and low plains. According to the Chinese system of soil texture classification, loam, clay soil, and sandy soil are the major soil types in Zhejiang Province. The province has a land area of 105,500 km^2^, of which approximately 81% is used for agricultural purposes [29]. Nearly 20% of the total area used as cultivated land, and the main agricultural products are cereals, soybeans, vegetables, tea, mulberries, and fruits. The rest of the agricultural land is used as garden land, woodland, grassland, and for other uses. The ratio of light industry to heavy industry is about 1:1. The major industries are manufacturing of products such as textiles, metal products, and plastic products [30].

This study evaluated heavy metal contamination in Zhejiang’s agricultural soils from 2016 to 2020. The study mainly focused on three metals (Pb, Cd, and Cr), all of which are listed as priority heavy metals for control by the Ministry of Ecology and Environment of the People’s Republic of China and the United States Environmental Protection Agency (U.S. EPA). We selected 20 administrative counties from 11 cities in Zhejiang Province as the research areas, and we used a random sampling method to select 20 administrative villages in five townships from each research area (Table S4).

### 2.2. Sample Collection and Measurement

During this study, 1999 samples of surface agricultural soils were taken at a depth of 20 cm. Each sample was composed of five random subsamples from each sampling point of 1 km^2^; the sample weight was about 1 kg. All samples were sealed in special polyethylene collection bags and transported back to the sample preparation laboratory within 12 h. The samples were dried at r oom temperature after organisms, debris, and stones were removed, ground with a polymethyl methacrylate stick and an agate grinder, sieved with a nylon sieve (2 mm; 10 mesh), sieved with another nylon sieve (0.25 mm; 60 mesh), processed with another nylon sieve (0.15 mm; 100 mesh), and mixed in a new sample collection bag for analysis.

Soil samples were weighed and placed in a polytetrafluoroethylene crucible. The method of acid digestion in a mixture of HCl-HNO_3_-HF-HClO_4_ (3:2:3:1 by volume) was used to digest the samples and completely integrate the heavy metals into the solution. An atomic absorption spectrometer (ZEEnit 700P, Analytik Jena AG, Jena, Germany) was employed to determine the concentrations of Pb, Cd, and Cr. Quality assurance and quality control were guaranteed with the soil standard reference materials (GBW07403, GBW07405 and GBW07406, the National Standard Detection Research Center, China). Analysis methods were validated by blanks, duplicates, and standard reference materials. The recovery of spiked standard ranged from 85% to 110%. The relative deviations of the duplicate samples were <7% for all batch treatments. The detection limits for Pb, Cd, and Cr were 0.1 mg·kg^−1^, 0.01 mg·kg^−1^, and 5 mg·kg^−1^, respectively.

### 2.3. Human Exposure and Health Risk Assessment

#### 2.3.1. Exposure Assessment

To assess the potential risk of Pb, Cd, and Cr to human health, different exposure routes of heavy metals in soils were explored, including oral ingestion, dermal absorption, and inhalation. The risk of exposure to heavy metals in soils was assessed with the U.S. EPA procedure and the exposure factors handbook for the Chinese population [31,32,33,34,35]. The assessment subjects were grouped into children under 18 years of age and adults. The average daily intake dose received through oral ingestion, dermal absorption, and inhalation was estimated using the following equations [1,32,36,37].

(1) $${\mathrm{ADI}}_{\mathrm{ingestion}}=\frac{C_{S}{\;\times \;\mathrm{IR}}_{\mathrm{ing}\;}\times \;\mathrm{ED}\;\times \;\mathrm{EF}\;\times \;\mathrm{FI}}{\mathrm{BW}\;\times \;\mathrm{AT}}{\times 10}^{–6}$$

(2) $${\mathrm{ADI}}_{\mathrm{dermal}}=\frac{C_{S}\;\times \;\mathrm{SA}\;\times \;\mathrm{AF}\;\times \;\mathrm{ABS}\;\times \;\mathrm{ED}\;\times \;\mathrm{EF}}{\mathrm{BW}\;\times \;\mathrm{AT}}{\times 10}^{–6}$$

(3) $${\mathrm{ADI}}_{\mathrm{inhalation}}=\frac{C_{S}{\;\times \;\mathrm{EF}\;\times \;\mathrm{ED}\;\times \;\mathrm{IR}}_{\mathrm{inh}}}{\mathrm{PEF}\;\times \;\mathrm{BW}\;\times \;\mathrm{AT}}$$

where ${\mathrm{ADI}}_{\mathrm{ingestion}}$, ${\mathrm{ADI}}_{\mathrm{dermal}}$, and ${\mathrm{ADI}}_{\mathrm{inhalation}}$ (mg/kg/day) are the average daily intake of heavy metals through oral ingestion, dermal absorption, and inhalation, respectively; Cs (mg/kg) is the concentration of heavy metals in soil. The definitions of the other variables are listed in Table S1.

#### 2.3.2. Non-Carcinogenic Risk Assessments

The hazard quotient (HQ) was used to calculate the non-carcinogenic risk, as proposed according to Equation (4).

(4) $$\mathrm{HQ}=\frac{\mathrm{ADI}}{\mathrm{RfD}}$$

where RfD (mg/kg/day) is the reference dose for different exposure pathways and metals (Table S2). 

To assess the total non-carcinogenic risk of multiple exposure pathways and metals, the Hazard Index (HI) statistic was calculated by Equation (5).

(5) HI=∑HQ

If HI is less than or equal to 1, there is no risk that non-carcinogenic effects are likely to occur. If HI is greater than 1, there is a high risk that non-carcinogenic effects are likely to occur.

#### 2.3.3. Carcinogenic Risk Assessments

Carcinogenic risk (CR), the possibility of an individual suffering from cancer due to exposure to carcinogenic risks, was calculated with the following equation:

(6) CR=ADI × SF

where SF (mg/kg/day) is the carcinogenic slope factor of different exposure routes and metals (Table S2). The range for generally acceptable risk is under 1 × 10^−4^.

### 2.4. Statistical Analysis

All data were statistically analyzed by the R software (version 4.0.4). The Kolmogorov–Smirnov (K–S) test was used to determine the normality of concentrations of heavy metals. Mean and standard deviation (SD) were used to describe normally distributed data, and non-normally distributed data were described by median and quartile. The Kruskal–Wallis Test was employed to analyze variations in soil heavy metal concentrations. The outliers were determined according to mean ± 3SD. The software used for mapping was ArcGIS 10.2 (ESRI, Redlands, CA, USA).

## 3. Results and Discussion

### 3.1. Levels of Heavy Metals in Agricultural Soils of Zhejiang Province

The median concentrations of Pb, Cd, and Cr in the agricultural soils in Zhejiang Province were 32.2 mg·kg^−1^, 0.18 mg·kg^−1^, and 42.3 mg·kg^−1^, respectively (Table 1). In comparison with the background values of Zhejiang Province, taken from a large sample from the national soil heavy metals survey in China [38], the average levels of Pb and Cd were higher, while the average level of Cr was slightly lower. Of the investigated samples, the concentrations of Pb, Cd, and Cr were 71.73%, 84.94%, and 36.92%, which exceeded the background value, respectively. The rates indicated that these three heavy metals may tend to accumulate in soils, with the highest accumulation of Cd, and the lowest accumulation of Cr. Although the average concentrations of these heavy metals were below the risk screening values [39], 27 Pb samples and 241 Cd samples still exceeded the risk screening values. The result indicated that contamination from these heavy metals of agricultural soils might induce potential risks to the public health in Zhejiang Province, especially Cd.

Overall, agricultural soils in Zhejiang Province were contaminated by heavy metals to varying extents, and Pb and Cd were accumulated to a certain degree. In comparison with other regions in China, the heavy metal levels in Zhejiang Province varied. The average concentration of Pb in Zhejiang Province was close to the medium concentration in the Yangtze River and Jianghan Plain, lower than that in the Pearl River Delta Region, and slightly higher than that in the northern region (Jilin, Hebei, and Beijing) [40,41,42,43,44,45]. The high concentration of Pb might be attributable to mining activities in Zhejiang. Pb-Zn mines are located in Wenzhou, Taizhou, and other cities and counties in Zhejiang, in which high concentrations of Pb are likely to be transferred from mining activities via atmospheric deposition and irrigation [25,46,47]. Besides, Pb was also likely arising from the legacy of lead-containing gasoline in the transportation sector [46,48,49,50,51,52]. 

The comparison of Cd with Pb exhibited a similar pattern, which might be related to agricultural activities. Previous studies have reported that Cd could accumulate through the application of chemical fertilizers, pesticides, and manure in agricultural production [46,53,54,55,56]. The use of fertilizers and pesticides likely contributes to higher Cd levels in agricultural soils. In addition, some researchers have indicated that Cd might originate from industrial activities such as coal burning and metal smelting [57,58,59,60]. There are several special industries in Zhejiang Province, such as e-waste dismantling, which might play an important role in transferring Cd to agricultural soils [61]. 

The average level of Cr in Zhejiang Province was much lower than that in other regions in China [62,63], and the concentrations at most sites were lower than the baseline concentrations, suggesting that Cr pollution in agricultural soils in Zhejiang is not obvious. Related studies have demonstrated that soil Cr might be derived from the process of soil formation and related to the weathering process of parent materials [64,65]. Mining, metal electroplating, tanning, and other human activities might release lower to higher Cr-containing effluents or solid wastes to the environment [66,67]. In recent years, related industries have shown downward trends in Zhejiang Province [30]. Moreover, some studies reported that typhoons might induce the redistribution of heavy metals in agricultural soils, which might be a reason for the lower concentrations [68]. Physical, chemical, and biological remediation processes might be potential tools for addressing the problem of Cr pollution [69,70].

### 3.2. Spatial Distribution and Temporal Trends of Heavy Metals

#### 3.2.1. Spatial Distribution

**Figure 2:**
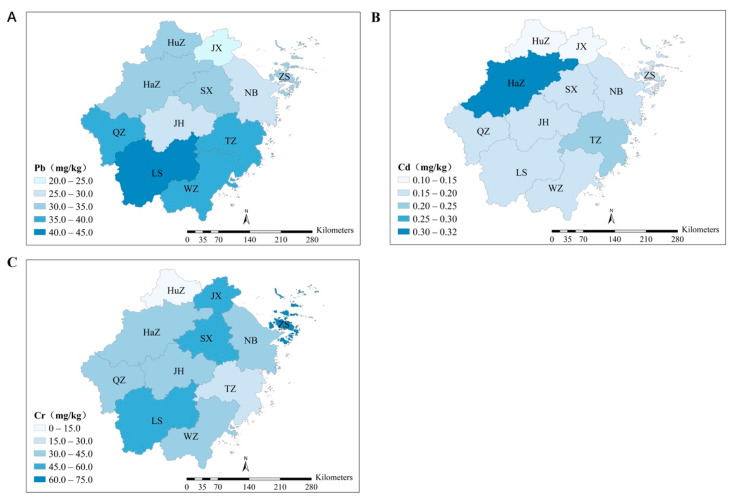
Average concentrations of heavy metals in agricultural soils in various cities in Zhejiang Province. (**A**): Average concentrations of Pb in agricultural soils in various cities in Zhejiang Province. (**B**): Average concentrations of Cd in agricultural soils in various cities in Zhejiang Province. (**C**): Average concentrations of Cr in agricultural soils in various cities in Zhejiang Province.

The levels of heavy metals in different sites in Zhejiang Province were assessed in this study (Figure 2, Table S3). The spatial distribution indicated that heavy metal concentrations had different regional characteristics in the investigated area. The findings revealed that agricultural soils in southern Zhejiang had higher Pb concentrations and the southwestern area had higher Cd concentrations, and there are no significant regional differences in the distribution of Cr concentration. The highest concentration of soil Pb was 40.9 mg·kg^−1^ and was detected in LS; the second-highest concentration of Pb was 37.6 mg·kg^−1^ and appeared in WZ. The higher Pb concentrations in southern Zhejiang might be associated with the geographical factors. This spatial distribution characteristic of Pb was similar to that of a previous survey on soil geochemical background values in Zhejiang Province [71]. In addition, the high concentration of Pb might be attributable to mining activities, such as Pb-Zn mines. Previous research found that the high soil Pb concentrations found in Zhejiang province were consistent with the location of the main Pb-Zn smelter in WZ [46]. 

The highest Cd concentration was observed in HaZ, which has a high degree of urbanization and industrialization. Fertilizers are reportedly important Cd sources for agricultural soils, and phosphatic fertilizers in HaZ constituted 13.14% of fertilizers used throughout the province, the second-highest concentrations; this finding is similar to the results of some previous studies [46]. Furthermore, the Cd concentration in TZ was higher than the rest of other cities. The reason for this situation might be related to e-waste dismantling activities because TZ is the largest e-waste dismantling sites in Zhejiang [72]. The concentrations of Cr were not an obvious spatial distribution characteristic. A large number of studies have shown that Cr mainly arises from soil parent material [46,49,50,73]. Sites with higher concentrations were generally found in ZS, which is one of the largest islands in China. Recent research indicated high levels of Cr near the Zhoushan Archipelago among the surface deposits of the Yangtze River and the adjacent East China Sea [74]. Furthermore, the concentrations of Cr in agricultural soil increased over time due to the excessive application of fertilizers and emissions from industrial activities [75,76]. Thus, the possible cause of the spatial distribution were local variations in industrial and agricultural development in Zhejiang Province.

#### 3.2.2. Temporal Trends

**Figure 3:**
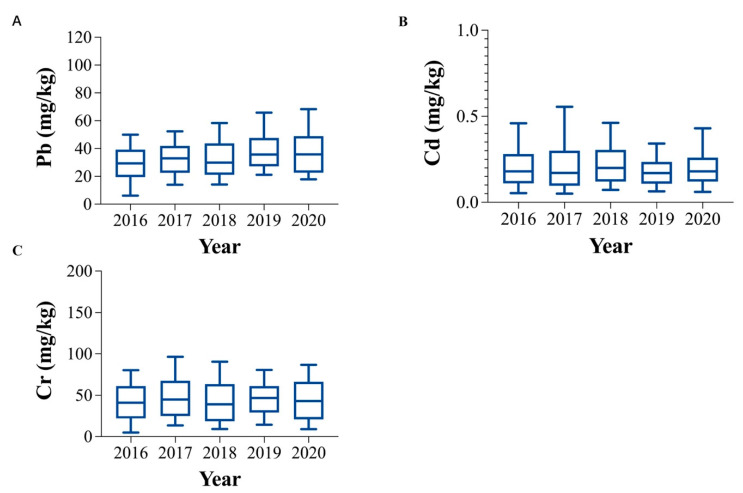
Temporal variations (2016–2020) in heavy metal concentrations (mg/kg) in agricultural soils in Zhejiang. (**A**): Temporal variations in Pb concentrations in agricultural soils in Zhejiang. (**B**): Temporal variations in Cd concentrations in agricultural soils in Zhejiang. (**C**): Temporal variations in Cr concentrations in agricultural soils in Zhejiang.

The temporal variation in the average heavy metal content showed various patterns in agricultural soils from 2016 to 2020 (Figure 3, Table S3). In terms of temporal distribution of Pb, the overall period showed a slow but gradual upward trend, except for a slight downward trend in 2018. The concentrations of Cd, and Cr showed slight fluctuations but no significantly temporal trend. In recent years, Chinese government has taken several measures to control the sources of pollution, such as the “Action Plan on Soil Pollution Prevention and Control (APSPPC)”, which was published in 2016. Industrial enterprises involved in potential soil pollution hazards were under control, which might be the reason for the stable concentration of Cd and Cr. However, heavy metals from agricultural activities were easily ignored. According to an analysis of the use of phosphate fertilizers in Zhejiang, the proportion was further increased since 2018, which might be one of the reasons for the turning point and upward trend of Pb [77]. The increasing rate of Pb transmission is a sign that if stricter regulation and remediation measures for Pb contamination are not implemented in the future, public safety in Zhejiang might be threatened. Recent research has shown that the application of phytoremediation technology could reduce the content of Pb in soil [78,79]. Therefore, necessary measures must be applied to enhance the transformation and regulation of industrial and agricultural activities to minimize further accumulation of heavy metals.

### 3.3. Human Health Risk Assessment

#### 3.3.1. Non-Carcinogenic Risk Assessment

**Figure 4:**
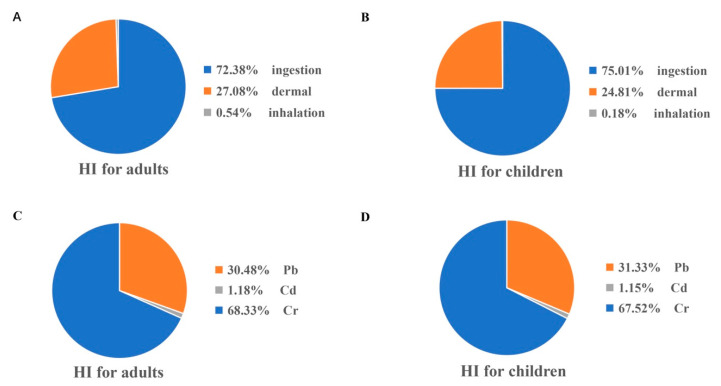
Contribution of various exposure routes and different metals to HI values. (**A**): Contribution of various routes to HI values for adults. (**B**): Contribution of various routes to HI values for children. (**C**): Contribution of different metals to HI values for adults. (**D**): Contribution of different metals to HI values for children.

To further analyze the potential threat to human health from the accumulation of heavy metals in agricultural soils, we conducted a human health risk assessment. The results for non-carcinogenic risks are shown in Table 2 and Figure 4. The total HI values for adults and children were 5.13 × 10^−2^ and 2.27 × 10^−1^, respectively. The HQs of these three heavy metals for children were all higher than those for adults. These results indicate that children are more susceptible to adverse effects due to the accumulation of heavy metals in agricultural soils, which may be the reason for special physiological, behavioral characteristics and vulnerability of some children. Overall, all of the HI values were less than 1, and negligible non-carcinogenic risks are likely to be experienced.

As shown in Figure 4A,B, the oral ingestion route contributed 72.38% of the non-carcinogenic risk for adults, followed by the dermal absorption route, which contributed 27.08%. For children, the oral ingestion route contributed 75.01%, and the dermal absorption route contributed 24.81%. The average oral ingestion HI for adults was 3.72 × 10^−2^, while the average HI of dermal absorption and inhalation were only 1.39 × 10^−2^ and 2.54 × 10^−4^, respectively. Similar proportions of these three routes regarding the non-carcinogenic risk for children and adults was also found. In general, oral ingestion is the major route of exposure to the health risks of heavy metals in the study area. Thus, attention should be paid to the importance of dietary exposure risk in the future, and activities should be scientifically implemented.

The average HI of Cr and Pb was higher than that Cd. The non-carcinogenic risks of adults and children due to Cr and Pb were mainly due to their high concentrations in soils or low reference doses. The average HQs of the three heavy metals decreased on the order of Cr > Pb > Cd (Figure 4C,D). The sum proportions of the average HI of Cr and Pb for adults and children were all close to 99%, which indicates that Cr and Pb cause the main non-carcinogenic effect factors of the three heavy metals in agricultural soils. Overall, the levels of heavy metals do not currently present a significant health risk to residents of Zhejiang province. Nevertheless, future effects due to the accumulation of Pb in soils cannot be ignored.

#### 3.3.2. Carcinogenic Risk Assessment

The assessment results for the carcinogenic risks of heavy metals in agricultural soils are presented in Table 2. The CR of Cd and Cr for all three exposure routes was calculated, whereas the carcinogenic risk of Pb was considered only from ingestion. Similar to the pattern of non-carcinogenic risk, the calculated CR values of the three heavy metals indicated higher carcinogenic risks for children than for adults. The average CR values of Pb, Cr, and Cd for children were 1.48 × 10^−7^, 6.02 × 10^−7^, and 1.68 × 10^−5^, respectively, and 1.70 × 10^−7^, 6.88 × 10^−7^, and 1.83 × 10^−5^, respectively, for adults. According to the CR values, carcinogenic risk is mainly due to the oral ingestion route, followed by dermal absorption. Relevant studies have shown that oral ingestion of metals might induce mild to severe gastrointestinal discomfort and tissue damage, and dermal absorption could cause contact dermatitis and other allergic responses [5]. As shown in Table 2, all of the CR values were under acceptable levels (1 × 10^−4^). However, that the average CR values for Cr were higher than those of the two other heavy metals for adults and children, which means a higher carcinogenic risk.

These results mainly indicate that children are more likely to face non-carcinogenic or carcinogenic risks from heavy metals from agricultural soils, which was consistent with other areas [61,80,81,82]. Children are more vulnerable to contamination by heavy metals due to their lower body weight and frequently special behavior [81,83]. Additionally, the HI and CR results in this study were lower than those found in other studies, which may be caused by the samples from non-industrial areas [61,72,84]. Thus, these findings might be more applicable to residents across the province, but the risks to residents near industrial areas might be underestimated. From the perspective of exposure routes, human health risks are mainly affected by ingestion routes. Cr and Pb are critical factors among the three heavy metals in agricultural soils. To reduce health risks from heavy metals in agricultural soils, rigid environmental regulations and soil remediation measures are necessary. For instance, related research indicated that the application of phytoremediation technology could improve the physical and chemical properties of soils, promote the remediation of soils, and reduce the health and environmental risks of heavy metals [78,79]. Besides, the application of the mixed fertilizers, soil amendment and enhancement could also effectively decrease the accumulation or lessen the bioavailability of heavy metals [61,85,86].

## 4. Conclusions

In this study, a total sum of 1999 agricultural soils from 11 cities in Zhejiang Province, China was analyzed from 2016 to 2020. The spatial distribution and temporal trends revealed that higher Pb concentrations were mainly found in southern Zhejiang, while higher Cd concentrations were found in the southwestern area. In addition, the overall temporal distribution of Pb showed a slow but gradual upward trend. Human health risk assessment showed that the major exposure route for heavy metals in agricultural soils is oral ingestion, and children are more susceptible to detrimental influence and effects. As for individual metals, Cr showed higher carcinogenic and non-carcinogenic risks than Pb and Cd. 

Although the health risk assessments of the studied areas were lower than those for industrial areas, enhanced environmental monitoring is still needed for some areas to ensure soil quality and protect human health by considering the persistence and bioaccumulation of heavy metals in the long term. Further cohort studies could be also carried out in some high heavy metal content agricultural sites to investigate their potentially adverse health effects from the perspective of molecular epidemiology.

## Figures and Tables

**Table 1:** Average concentration (mg·kg^−1^) of heavy metals in agricultural soils in Zhejiang Province and other regions.

Year	Location	Sample Numbers	Pb			Cd			Cr			Reference
			Median	Min	Max	Median	Min	Max	Median	Min	Max	
2019	Yangtze River, China	95	29.30	14.20	720.00	0.27	0.12	1.74	84.40	61.60	119.00	[31]
2016	Jilin, China	79	23.42	7.00	70.35	0.155	0.046	0.620	-	-	-	[33]
2016	Guanzhong Plain, China	227	24.4	16.8	125.1	-	-	-	69.4	55.5	306.2	[53]
2015	Jianghan Plain, China	234	34.3	3.40	68.9	0.45	0.06	1.46	-	-	-	[32]
2012	Hebei, China	287	23.50	13.70	125.70	0.16	0.05	4.52	67.20	25.00	112.10	[34]
2009	Beijing, China	412	20.4	10.3	37.5	0.136	0.015	0.469	-	-	-	[35]
2008	Huanghuai Plain, China	224	24.0	14.3	28.6	0.16	0.06	0.52	72.2	47.7	361	[54]
2002–2005	Pearl River Delta, China	1384	46.69	7.06	284	0.14	0.004	2.95	39.5	0.72	1137	[36]
2016–2020	Zhejiang, China	1999	32.2	0.05	151.0	0.18	0.005	2.40	42.3	2.5	237.4	This study
1990	Zhejiang background value, China	76	23.7	-	-	0.07	-	-	52.9	-	-	[29]
1990	Background value, China	4095	26.0	-	-	0.097	-	-	61.0	-	-	[29]

**Table 2:** Health risk assessment of different routes and heavy metals.

	Non–Carcinogenic Hazard Index		Carcinogenic Risk Index	
	Mean for Adults	Mean for Children	Mean for Adults	Mean for Children
Ingestion	3.72 × 10^−2^	1.70 × 10^−1^	1.22 × 10^−5^	1.40 × 10^−5^
Dermal	1.39 × 10^−2^	5.62 × 10^−2^	5.15 × 10^−6^	5.20 × 10^−6^
Inhalation	2.75 × 10^−4^	4.05 × 10^−4^	1.42 × 10^−7^	3.10 × 10^−8^
Pb	1.56 × 10^−2^	7.10 × 10^−2^	1.48 × 10^−7^	1.70 × 10^−7^
Cd	6.07 × 10^−4^	2.60 × 10^−3^	6.02 × 10^−7^	6.88 × 10^−7^
Cr	3.51 × 10^−2^	1.53 × 10^−1^	1.68 × 10^−5^	1.83 × 10^−5^
Total	5.13 × 10^−2^	2.27 × 10^−1^	1.75 × 10^−5^	1.92 × 10^−5^

## Data Availability

The data presented in this study are available on request from the corresponding author.

## Supplementary Materials

The following supporting information can be downloaded at: https://www.mdpi.com/article/10.3390/ijerph192214642/s1, Table S1: Parameters used for the health risk assessment in this study; Table S2: Reference dose (*RfD*) and slope factor (*SF*) of heavy metals for health risk assessment; Table S3: The concentration of heavy metals in agricultural soils of different cities from 2016 to 2020 (mg·kg^−1^); Table S4: Detailed sampling locations in this study. References [33,34,35,87,88,89,90,91,92,93] are cited in the supplementary materials.
