# Supplementary material for: Pollution Characteristics and Health Risk Assessment of Heavy Metals in Agricultural Soils over the Past Five Years in Zhejiang, Southeast China
Source: Int J Environ Res Public Health. 2022 Nov 8;19(22):14642. doi: 10.3390/ijerph192214642 (PMC9690052; doi:10.3390/ijerph192214642)
Supplement: Supplementary file 1 [file ijerph-19-14642-s001.zip › ijerph-1987600-supplementary.pdf]

Supplemental Materials

Pollution Characteristics and Health Risk Assessment of Heavy Metals in Agricultural Soils over the Past Five Years in Zhejiang, Southeast China

Jie Xiang [1,†], Peiwei Xu [1,†], Weizhong Chen [1], Xiaofeng Wang [1], Zhijian Chen [1], Dandan Xu [1], Yuan Chen [1], Mingluan Xing [1], Ping Cheng [1], Lizhi Wu [1,*] and Bing Zhu [2,*]

[1] Department of Environmental Health, Zhejiang Provincial Center for Disease Control and Prevention, Hangzhou 310000, China
[2] Hangzhou Center for Disease Control and Prevention, Hangzhou 310000, China
* Correspondence: lzhwu@cdc.zj.cn (L.W.); 96zhubing@163.com (B.Z.); Tel.: +86–0571–87115221 (L.W.); +86–13588012016 (B.Z.)
† These authors contributed equally to this work.

Table S1. Parameters used for the health risk assessment in this study.

Parameters	Description	Units	Value	References
IR_{ing}	ingestion rate of soil	mg/day	100 for adults<br>200 for children	[1,2]
IR_{inh}	inhalation rate of soil	m^3/day	15.7 for adults<br>10.1 for children	[3,4]
ED	exposure duration	year	24 for adults<br>6 for children	[1,2]
EF	exposure frequency	days/year	350	[1,2]
FI	factor ingested from contamination source	unitless	1	
BW	body weight of an exposed individual	kg	60.6 for adults<br>26.5 for children	[3,4]
AT	average exposure time	days	ED × 365 for non-carcinogens<br>70 × 365 for carcinogens	[1,2]
SA	exposed skin surface area	cm^2	16000 for adults<br>9900 for children	[3,4]
AF	adherence factor	mg/cm^2 day	0.07 for adults<br>0.2 for children	[5,6]
ABS	dermal absorption factor	unitless	0.001	[1]
PEF	emission factor	m^3/kg	1.36×10^9	[7]

Table S2. Reference dose (*RfD*) and slope factor (*SF*) of heavy metals for health risk assessment.

	RfD for ingestion	*RfD* for dermal absorption	*RfD* for inhalation	*SF* for ingestion	*SF* for dermal absorption	*SF* for inhalation	Reference
Pb	0.0035	0.000525	0.00352	0.0085	-	-	[8,9]
Cd	0.001	0.00001	0.00001	6.1	6.1	6.3	[8,9]
Cr	0.003	0.00006	0.0000286	0.5	20	42	[8–10]

Table S3 The concentration of heavy metals in agricultural soils of different cities from 2016 to 2020 ($mg \cdot kg^{-1}$).

Year	Metal	N	Zhejiang province	City										
				HaZ	NB	WZ	JX	HuZ	SX	JH	QZ	ZS	TZ	LS
2016	Pb	381	29.3 (20.3,39.2)	39.3 ± 12.5	34.9 ± 8.6	4.5 (3.6,4.8)	29.2 (24.2, 32.9)	50.8 ± 17.5	32.2 (27.2, 36.6)	33.6 ± 17.4	20.5 ± 8.6	41.2 (29.5,52.7)	28.3 ± 10.1	21.5 (14.8,38.8)
	Cd	381	0.18 (0.11,0.28)	0.16 (0.09,0.34)	0.18 (0.15,0.21)	0.30 (0.22,0.34)	0.09 (0.06,0.19)	0.10 ± 0.10	0.23 (0.14,0.34)	0.18 (0.10,0.40)	0.16 (0.12,0.22)	0.18 (0.14,0.23)	0.16±0.06	0.29 (0.15,0.50)
	Cr	381	41.3 (23.8,61.1)	40.6 ± 18.7	37.2 (26.3,42.7)	29.4 ± 9.3	56.9 ± 10.4	2.5 (2.5,7.0)	34.8 ± 19.6	63.2 (39.4,90.4)	44.6 (27.4,58.9)	67.6 ± 6.2	76.0 ± 26.6	4.1 (2.1,7.6)
2017	Pb	372	32.6±15.1	37.9 (34.0,40.4)	39.6 ± 12.8	31.4 (5.6,45.7)	21.6 (17.4,28.2)	30.8 ± 6.8	31.1 (26.0,39.8)	36.7 ± 12.5	18.9 ± 8.7	34.4 ± 12.6	40.3 ± 10.3	34.4 ± 18.4
	Cd	372	0.17 (0.09,0.30)	0.35 (0.25,0.61)	0.15 (0.10,0.22)	0.22 (0.21,0.26)	0.06 (0.03,0.11)	0.17 (0.09,0.41)	0.17 (0.11,0.47)	0.12 (0.08,0.24)	0.14 (0.10,0.18)	0.09±0.06	0.19 (0.14,0.32)	0.17 (0.08,0.37)
	Cr	372	45.0 (25.0,67.7)	52.0 ± 21.0	60.1 ± 27.6	41.3 (24.3,54.7)	68.1 (37.1,96.8)	56.0 (47.0,58.0)	34.2 (17.8,42.3)	51.1 (22.8,89.7)	37.9 (21.4,64.2)	58.3 ± 24.2	63.6 (36.2,146.7)	6.9 (4.0,14.9)
2018	Pb	388	29.4 (20.7,43.3)	29.3 (25.5,33.5)	31.1 (26.8,40.3)	40.0 ± 12.1	19.1 ± 5.7	27.9 (24.5,34,9)	32.5 (23.0,56.7)	66.0 (31.6,83.8)	13.4 (11.3,33.0)	28.3 ± 17.3	39.4 ± 9.9	23.8 (16.2,43.4)
	Cd	388	0.20 (0.12,0.31)	0.47 ± 0.28	0.16 (0.12,0.20)	0.27 (0.19,0.37)	0.13 (0.09,0.31)	0.28 (0.15,0.42)	0.22 ± 0.14	0.14 (0.08,0.33)	0.12 ± 0.10	0.21 (0.18,0.24)	0.24 ± 0.09	0.17 (0.12,0.26)
	Cr	388	39.2 (18.4,63.5)	48.1 (22.3,64.3)	63.5±25.1	22.5 (11.5,47.3)	37.1 (22.2,63.9)	24.6 ± 9.7	45.1 (12.1,60.7)	41.8 (38.5,52.5)	26.1 ± 17.8	42.4 ± 35.5	107.6 ± 34.0	13.5 (7.2,24.9)
2019	Pb	383	35.6 (27.2,47.6)	36.0 (29.7,42.8)	29.1 (24.1,39.7)	46.7 (41.6,55.0)	26.8 ± 7.0	24.0 ± 4.4	56.9 (38.6,66.1)	35.8 (30.6, 46.3)	39.8 (36.1,52.4)	76.3 ± 24.3	22.3 (17.2,29.8)	37.0 ± 12.7
	Cd	383	0.17 (0.11,0.23)	0.38 ± 0.19	0.20 (0.17,0.24)	0.20 ± 0.09	0.14 (0.10,0.19)	0.19 ± 0.04	0.17 ± 0.09	0.15 (0.10,0.20)	0.21 (0.15,0.31)	0.01 (0.01,0.09)	0.15 ± 0.07	0.11 (0.05,0.13)

	Cr	383	46.0 (29.1,61.1)	40.0 (31.4,46.0)	68.8 ± 11.8	32.4 (2.5,63.2)	37.2 (32.5,51.0)	53.0 ± 14.6	52.4 (47.0,59.2)	31.3 (23.0,47.1)	31.5 ± 14.0	50.9 ± 13.1	45.6 (6.3,110.0)	51.0 (41.8,58.8)
2020	Pb	386	35.2 (22.5,48.6)	27.2 (21.0,45.3)	22.2 (17.8,37.7)	39.2 (25.7,52.0)	23.2 (21.5,26.9)	24.1 (18.8,29.6)	51.9 (27.9,65.5)	48.0 (16.0,54.3)	36.2 (18.0,48.8)	45.3 (40.9,54.0)	36.5 (30.4,44.6)	42.5 ± 10.9
	Cd	386	0.18 (0.12,0.26)	0.23 (0.13,0.51)	0.18 (0.12,0.23)	0.23 ± 0.10	0.16 (0.13,0.20)	0.19 ± 0.12	0.17 (0.12,0.22)	0.25 (0.06,0.60)	0.20 (0.11,0.34)	0.17 ± 0.05	0.16 (0.07,0.27)	0.15 (0.05,0.21)
	Cr	386	42.0 (20.6, 66.4)	45.7 ± 22.1	68.5 (29.0,82.5)	19.0 (9.2,48.6)	72.8 ± 16.3	48.0 ± 13.0	47.4 ± 18.8	9.5 (3.4,49.9)	51.0 ± 20.3	30.0 (25.3,42.8)	35.0 (15.0,83.0)	15.6 (9.7,28.7)

Table S4. Detailed sampling locations in this study

Number	Longitude	Latitude	Number	Longitude	Latitude	Number	Longitude	Latitude	Number	Longitude	Latitude
1	119.58	29.83	501	120.56	27.59	1001	120.75	29.53	1501	122.41	29.93
2	119.61	29.81	502	120.55	27.61	1002	120.70	29.51	1502	122.41	29.91
3	119.58	29.82	503	120.54	27.62	1003	120.68	29.49	1503	122.40	29.92
4	119.61	29.80	504	120.57	27.61	1004	120.75	29.54	1504	122.40	29.91
5	119.61	29.70	505	120.41	27.58	1005	120.71	29.58	1505	122.29	30.03
6	119.61	29.69	506	120.41	27.57	1006	120.72	29.65	1506	122.30	30.03
7	119.64	29.72	507	120.40	27.57	1007	120.77	29.64	1507	122.29	30.03
8	119.71	29.66	508	120.45	27.59	1008	120.65	29.60	1508	122.27	30.05
9	119.83	29.76	509	120.56	27.72	1009	120.77	29.54	1509	122.10	29.74
10	119.81	29.74	510	120.56	27.71	1010	120.80	29.60	1510	122.14	29.76
11	119.76	29.80	511	120.58	27.71	1011	120.78	29.59	1511	122.14	29.70
12	119.78	29.80	512	120.59	27.70	1012	120.80	29.54	1512	122.11	29.78
13	119.54	29.90	513	120.38	27.41	1013	120.86	29.62	1513	122.27	29.84
14	119.54	29.90	514	120.65	27.69	1014	120.86	29.64	1514	122.30	29.82
15	119.45	29.84	515	120.64	27.70	1015	120.88	29.64	1515	122.29	29.84
16	119.54	29.90	516	120.63	27.70	1016	120.89	29.61	1516	122.26	29.83
17	119.46	29.94	517	120.31	27.65	1017	120.70	29.45	1517	122.24	29.76
18	119.46	29.94	518	120.30	27.62	1018	120.72	29.45	1518	122.23	29.78
19	119.44	29.95	519	120.38	27.66	1019	120.69	29.44	1519	122.26	29.78
20	119.40	29.91	520	120.32	27.62	1020	120.68	29.43	1520	122.25	29.76
21	119.16	29.85	521	120.78	30.39	1021	119.79	28.95	1521	121.49	28.62
22	119.07	29.86	522	120.81	30.36	1022	119.77	28.98	1522	121.50	28.64
23	119.13	29.89	523	120.79	30.36	1023	119.76	28.92	1523	121.46	28.62
24	119.15	29.81	524	120.78	30.36	1024	119.76	28.94	1524	121.46	28.62
25	118.76	29.41	525	120.76	30.46	1025	119.70	28.73	1525	121.41	28.60
26	118.80	29.37	526	120.77	30.45	1026	119.85	28.75	1526	121.41	28.60
27	118.80	29.32	527	120.79	30.41	1027	119.78	28.74	1527	121.41	28.61
28	118.77	29.33	528	120.79	30.42	1028	119.76	28.66	1528	121.41	28.63
29	118.79	29.41	529	120.64	30.53	1029	119.60	28.82	1529	121.39	28.73
30	118.78	29.33	530	120.67	30.75	1030	119.66	28.89	1530	121.40	28.73
31	118.77	29.82	531	120.70	30.55	1031	119.65	28.83	1531	121.41	28.72
32	118.82	29.81	532	120.71	30.53	1032	119.65	28.82	1532	121.42	28.72
33	118.66	29.51	533	120.57	30.45	1033	119.61	28.57	1533	121.44	28.71
34	118.71	29.51	534	120.59	30.46	1034	119.64	28.55	1534	121.46	28.71
35	118.73	29.51	535	120.58	30.45	1035	119.64	28.56	1535	121.49	28.73
36	118.53	29.56	536	120.56	30.44	1036	119.69	28.55	1536	121.49	28.72
37	118.56	29.44	537	120.58	30.51	1037	119.63	28.74	1537	121.37	28.65
38	118.55	29.43	538	120.57	30.51	1038	119.66	28.72	1538	121.40	28.63
39	118.58	29.47	539	120.55	30.48	1039	119.66	28.73	1539	121.41	28.65
40	118.51	29.40	540	120.56	30.49	1040	119.63	28.71	1540	121.40	28.66
41	121.95	29.56	541	120.52	30.58	1041	119.46	29.27	1541	120.46	28.71

42	121.94	29.57	542	120.49	30.59	1042	119.45	29.28	1542	120.50	28.76
43	121.93	29.57	543	120.49	30.60	1043	119.46	29.28	1543	120.48	28.65
44	121.92	29.56	544	120.44	30.58	1044	119.44	29.31	1544	120.40	28.67
45	121.84	29.24	545	120.59	30.71	1045	119.41	29.18	1545	120.64	28.79
46	121.85	29.23	546	120.56	30.69	1046	119.40	29.18	1546	120.63	28.78
47	121.88	29.24	547	120.52	30.71	1047	119.37	29.17	1547	120.63	28.78
48	121.81	29.24	548	120.55	30.73	1048	119.38	29.21	1548	120.65	28.79
49	121.78	29.58	549	120.36	30.49	1049	119.48	29.13	1549	120.66	28.83
50	121.80	29.58	550	120.36	30.49	1050	119.50	29.15	1550	120.67	28.83
51	121.82	29.58	551	120.35	30.52	1051	119.50	29.16	1551	120.67	28.84
52	121.81	29.60	552	120.37	30.53	1052	119.47	29.16	1552	120.67	28.86
53	121.79	29.42	553	120.36	30.57	1053	119.48	29.25	1553	120.54	28.75
54	121.83	29.41	554	120.35	30.57	1054	119.47	29.23	1554	120.53	28.76
55	121.85	29.39	555	120.35	30.57	1055	119.49	29.22	1555	120.52	28.76
56	121.81	29.38	556	120.34	30.58	1056	119.49	29.23	1556	120.53	28.78
57	121.89	29.42	557	120.53	30.49	1057	119.35	29.36	1557	120.60	28.74
58	121.89	29.41	558	120.55	30.50	1058	119.41	29.34	1558	120.60	28.76
59	121.87	29.38	559	120.54	30.55	1059	119.41	29.32	1559	120.59	28.77
60	121.88	29.34	560	120.56	30.52	1060	119.41	29.31	1560	120.61	28.74
61	121.33	30.01	561	119.25	30.33	1061	118.22	29.23	1561	119.38	28.64
62	121.32	30.01	562	119.39	30.58	1062	118.22	29.23	1562	119.36	28.61
63	121.34	30.03	563	119.38	30.55	1063	118.23	29.19	1563	119.36	28.64
64	121.34	30.03	564	119.20	30.31	1064	118.25	29.18	1564	119.37	28.65
65	121.06	29.90	565	119.47	30.46	1065	118.52	29.14	1565	118.99	28.36
66	121.91	29.07	566	119.46	30.48	1066	118.50	29.14	1566	118.98	28.35
67	121.06	29.90	567	119.47	30.46	1067	118.47	29.11	1567	118.98	28.37
68	121.06	29.90	568	119.46	30.48	1068	118.45	29.10	1568	119.02	28.35
69	120.99	30.08	569	119.65	30.83	1069	118.44	29.05	1569	119.06	28.51
70	121.15	30.04	570	119.56	30.82	1070	118.35	29.01	1570	119.05	28.48
71	120.98	30.08	571	119.60	30.82	1071	118.35	29.03	1571	119.07	28.51
72	121.00	30.08	572	119.65	30.83	1072	118.37	29.06	1572	119.04	28.48
73	121.06	30.20	573	119.37	30.31	1073	118.40	29.33	1573	119.06	28.71
74	121.05	30.23	574	119.36	30.31	1074	118.39	29.30	1574	119.07	28.72
75	121.08	30.24	575	119.37	30.31	1075	118.38	29.27	1575	119.10	28.73
76	121.06	30.23	576	119.39	30.31	1076	118.37	29.26	1576	119.04	28.69
77	121.17	30.15	577	119.57	30.67	1077	118.36	29.21	1577	119.19	28.44
78	121.14	30.14	578	119.57	30.64	1078	118.36	29.19	1578	119.17	28.41
79	121.18	30.16	579	119.52	30.70	1079	118.38	29.18	1579	119.12	28.40
80	121.20	30.15	580	119.56	30.68	1080	118.41	29.23	1580	119.18	28.35
81	120.72	28.46	581	120.34	29.84	1081	118.71	28.79	1581	119.34	28.06
82	120.71	28.48	582	120.33	29.92	1082	118.69	28.79	1582	119.32	28.06
83	120.71	28.48	583	120.64	29.91	1083	118.74	28.77	1583	119.96	27.86
84	120.73	28.45	584	120.33	29.92	1084	118.70	28.78	1584	119.36	28.07

85	120.76	28.32	585	120.19	29.58	1085	118.45	28.62	1585	118.99	28.03
86	120.82	28.32	586	120.15	29.59	1086	118.47	28.63	1586	119.02	28.04
87	120.75	28.31	587	120.11	29.39	1087	118.45	28.63	1587	119.00	28.02
88	120.77	28.31	588	120.15	29.59	1088	118.47	28.63	1588	119.27	27.88
89	120.69	28.07	589	120.42	29.79	1089	118.50	28.55	1589	118.99	27.88
90	120.69	28.07	590	120.38	29.74	1090	118.81	28.87	1590	118.99	27.88
91	120.69	28.08	591	120.46	29.83	1091	118.50	28.57	1591	118.99	27.88
92	120.69	28.11	592	120.44	29.77	1092	118.46	28.56	1592	118.99	27.88
93	120.57	28.31	593	120.27	29.64	1093	118.46	28.70	1593	119.10	27.98
94	120.56	28.32	594	120.28	29.64	1094	118.46	28.69	1594	119.08	28.00
95	120.56	28.31	595	120.29	29.62	1095	118.49	28.65	1595	119.10	27.98
96	120.56	28.31	596	120.27	29.63	1096	118.46	28.66	1596	119.10	27.98
97	120.78	28.23	597	120.15	29.89	1097	118.88	28.89	1597	119.04	28.08
98	120.76	28.20	598	120.13	29.85	1098	118.69	28.84	1598	119.03	28.06
99	120.75	28.21	599	120.20	29.90	1099	118.69	28.90	1599	119.02	28.08
100	120.75	28.18	600	120.21	29.88	1100	118.68	28.85	1600	119.01	28.08
101	120.66	27.66	601	120.74	29.53	1101	121.89	30.05	1601	119.60	30.12
102	120.64	27.66	602	120.70	29.51	1102	121.89	30.02	1602	119.53	30.11
103	120.64	27.66	603	120.68	29.49	1103	121.86	30.04	1603	119.58	30.10
104	120.64	27.68	604	120.75	29.54	1104	121.87	30.01	1604	119.58	30.17
105	120.55	27.61	605	120.71	29.58	1105	122.14	30.08	1605	120.01	29.90
106	120.54	27.60	606	120.72	29.65	1106	122.18	30.07	1606	120.00	29.90
107	120.49	27.60	607	120.77	29.64	1107	122.17	30.07	1607	119.98	29.90
108	120.48	27.60	608	120.65	29.60	1108	122.17	30.09	1608	120.00	29.89
109	120.26	27.62	609	120.77	29.54	1109	122.12	30.09	1609	119.89	29.89
110	120.26	27.61	610	120.80	29.60	1110	122.16	30.11	1610	119.91	29.88
111	120.25	27.60	611	120.78	29.59	1111	122.13	30.11	1611	119.88	29.89
112	120.21	27.58	612	120.80	29.54	1112	122.14	30.12	1612	119.92	29.89
113	120.31	27.65	613	120.86	29.64	1113	122.05	30.05	1613	119.92	30.02
114	120.31	27.65	614	120.90	29.64	1114	122.03	30.05	1614	119.92	30.02
115	120.34	27.65	615	120.89	29.63	1115	122.02	30.05	1615	119.89	29.99
116	120.34	27.65	616	120.88	28.64	1116	122.03	30.06	1616	119.91	30.01
117	120.23	27.64	617	120.60	29.36	1117	122.08	30.13	1617	119.81	30.10
118	120.23	27.65	618	120.62	29.41	1118	122.09	30.12	1618	119.86	30.06
119	120.24	27.63	619	120.61	29.41	1119	122.09	30.12	1619	119.83	30.06
120	120.27	27.64	620	120.63	29.36	1120	122.08	30.13	1620	119.83	30.11
121	120.46	30.23	621	119.81	28.93	1121	121.21	28.09	1621	119.69	30.33
122	120.49	30.21	622	119.79	28.88	1122	121.22	28.10	1622	119.68	30.37
123	120.47	30.21	623	119.73	28.95	1123	121.21	28.10	1623	119.63	30.40
124	120.47	30.21	624	119.74	28.89	1124	121.20	28.10	1624	119.66	30.38
125	120.46	30.27	625	119.76	28.76	1125	121.17	28.14	1625	119.80	30.15
126	120.46	30.27	626	119.77	28.86	1126	121.20	28.18	1626	119.84	30.19
127	120.47	30.24	627	119.77	28.79	1127	121.19	28.17	1627	119.81	30.19

128	120.47	30.25	628	119.76	28.83	1128	121.17	28.15	1628	119.79	30.19
129	120.38	30.31	629	119.67	28.80	1129	121.19	28.11	1629	119.46	30.19
130	120.40	30.32	630	119.67	28.79	1130	121.21	28.09	1630	119.48	30.20
131	120.42	30.32	631	119.66	28.77	1131	121.18	28.10	1631	119.48	30.20
132	128.42	30.32	632	119.62	28.77	1132	121.18	28.12	1632	119.52	30.17
133	120.33	30.27	633	119.86	28.88	1133	121.13	28.11	1633	119.30	30.19
134	120.35	30.27	634	119.88	28.87	1134	121.15	28.12	1634	119.31	30.19
135	120.34	30.27	635	119.86	28.84	1135	121.14	28.11	1635	119.31	30.21
136	120.33	30.26	636	119.89	28.85	1136	121.12	28.11	1636	119.33	30.19
137	120.34	30.30	637	119.56	28.63	1137	121.12	28.05	1637	118.95	30.31
138	120.34	30.30	638	119.57	28.65	1138	121.22	28.09	1638	118.97	30.31
139	120.33	30.28	639	119.54	28.71	1139	121.18	28.06	1639	119.00	30.30
140	120.33	30.29	640	119.57	28.67	1140	121.20	28.06	1640	118.96	30.30
141	120.55	30.69	641	119.61	29.32	1141	121.17	28.08	1641	121.37	29.28
142	120.49	30.68	642	119.58	29.31	1142	121.04	29.24	1642	121.37	29.28
143	120.49	30.66	643	119.58	29.30	1143	121.03	29.24	1643	121.35	29.28
144	120.46	30.67	644	119.59	29.29	1144	121.05	29.24	1644	121.38	29.29
145	120.50	30.53	645	119.53	29.28	1145	121.21	29.06	1645	121.67	29.33
146	120.48	30.52	646	119.52	29.29	1146	121.20	29.05	1646	121.69	29.32
147	120.48	30.50	647	119.54	29.27	1147	121.20	29.05	1647	121.69	29.38
148	120.42	30.54	648	119.53	29.27	1148	121.20	29.06	1648	121.68	29.30
149	120.37	30.65	649	119.43	29.20	1149	121.14	29.11	1649	121.59	29.31
150	120.38	30.65	650	119.51	29.21	1150	121.16	29.11	1650	121.57	29.31
151	120.40	30.63	651	119.51	29.19	1151	121.12	29.11	1651	121.54	29.34
152	120.42	30.64	652	119.52	29.17	1152	121.11	29.12	1652	121.59	29.32
153	120.53	30.74	653	119.39	29.24	1153	120.79	29.11	1653	121.29	29.13
154	120.52	30.72	654	119.29	29.22	1154	120.79	29.11	1654	121.29	29.13
155	120.52	30.71	655	119.29	29.23	1155	120.80	29.12	1655	121.32	29.18
156	120.48	30.72	656	119.30	29.25	1156	120.80	29.12	1656	121.33	29.16
157	120.63	30.59	657	119.30	29.24	1157	120.94	29.24	1657	121.35	29.45
158	120.59	30.55	658	119.33	29.24	1158	120.94	29.23	1658	121.31	29.41
159	120.60	30.56	659	119.36	29.21	1159	120.95	29.23	1659	121.34	29.41
160	120.60	30.57	660	119.35	29.22	1160	121.93	29.24	1660	121.30	29.45
161	119.43	30.56	661	118.44	29.08	1161	120.10	28.70	1661	121.36	30.16
162	119.38	30.57	662	118.44	29.06	1162	120.07	28.70	1662	121.37	30.17
163	119.36	30.56	663	118.44	29.05	1163	120.11	28.66	1663	121.34	30.17
164	119.34	30.52	664	118.44	29.05	1164	120.13	28.67	1664	121.37	30.15
165	119.78	30.78	665	118.43	29.24	1165	120.06	28.68	1665	121.30	30.23
166	119.78	30.81	666	118.41	29.23	1166	120.05	28.68	1666	121.32	30.24
167	119.77	30.77	667	118.39	29.21	1167	120.04	28.68	1667	121.31	30.23
168	119.77	30.79	668	118.38	29.19	1168	120.06	28.68	1668	121.30	30.23
169	119.62	30.83	669	118.45	29.27	1169	120.13	28.66	1669	121.30	30.19
170	119.57	30.82	670	118.46	29.26	1170	120.16	28.65	1670	121.32	30.18

171	119.57	30.81	671	118.51	29.30	1171	120.16	28.65	1671	121.30	30.19
172	119.65	30.84	672	118.50	29.29	1172	120.20	28.66	1672	121.31	30.18
173	119.63	30.53	673	118.12	29.19	1173	120.16	28.75	1673	121.13	30.22
174	119.60	30.53	674	118.11	29.16	1174	120.16	28.76	1674	121.12	30.20
175	119.68	30.52	675	118.13	29.18	1175	120.17	28.76	1675	121.12	30.20
176	119.67	30.50	676	118.11	29.22	1176	120.17	28.75	1676	121.12	30.20
177	119.56	30.67	677	118.25	29.00	1177	120.26	28.51	1677	121.37	30.27
178	119.56	30.64	678	118.22	29.03	1178	120.27	28.53	1678	121.32	30.26
179	119.51	30.69	679	118.23	29.02	1179	120.26	28.52	1679	121.35	30.27
180	119.55	30.68	680	118.25	29.00	1180	120.24	28.52	1680	121.33	30.25
181	120.46	29.76	681	118.51	28.65	1181	119.39	28.51	1681	120.63	27.90
182	120.45	29.74	682	118.52	28.64	1182	119.40	28.50	1682	120.62	27.90
183	120.48	29.78	683	118.56	28.66	1183	119.39	28.50	1683	120.64	27.90
184	120.44	29.73	684	118.50	28.66	1184	119.41	28.49	1684	120.62	27.90
185	120.22	29.64	685	118.66	28.59	1185	119.43	28.39	1685	120.47	28.00
186	120.22	29.65	686	118.66	28.60	1186	119.43	28.40	1686	120.48	28.02
187	120.24	29.64	687	118.65	28.59	1187	119.45	28.36	1687	120.47	28.02
188	120.21	29.67	688	118.60	28.61	1188	119.44	28.40	1688	120.48	28.01
189	120.49	29.43	689	118.64	28.77	1189	119.64	28.35	1689	120.67	27.88
190	120.49	29.43	690	118.70	28.81	1190	119.68	28.34	1690	120.67	27.88
191	120.50	29.43	691	118.73	28.79	1191	119.65	28.36	1691	120.67	27.89
192	120.49	29.43	692	118.70	28.79	1192	119.68	28.34	1692	120.67	27.88
193	120.33	29.57	693	118.53	28.45	1193	119.38	28.54	1693	120.93	27.96
194	120.34	29.56	694	118.51	28.44	1194	119.38	28.55	1694	120.59	27.95
195	120.31	29.61	695	118.49	28.49	1195	119.76	28.56	1695	120.60	27.94
196	120.27	29.56	696	118.54	28.60	1196	119.39	28.67	1696	120.62	27.95
197	120.34	29.88	697	118.29	28.70	1197	119.52	28.36	1697	120.70	27.92
198	120.40	29.94	698	115.03	27.07	1198	119.45	28.32	1698	120.71	27.92
199	120.38	29.92	699	118.53	28.77	1199	119.45	28.32	1699	120.71	27.91
200	120.33	29.93	700	118.53	28.73	1200	119.44	28.31	1700	120.70	27.91
201	120.86	29.64	701	118.90	28.94	1201	120.00	30.00	1701	120.69	28.08
202	120.90	29.64	702	122.21	30.30	1202	120.00	30.00	1702	120.69	28.11
203	120.89	29.63	703	122.22	30.31	1203	120.00	30.00	1703	120.69	28.08
204	120.88	28.64	704	122.22	30.32	1204	120.00	30.00	1704	120.68	28.07
205	120.74	29.53	705	122.14	30.31	1205	119.00	29.00	1705	120.76	28.20
206	120.70	29.51	706	122.18	30.30	1206	119.00	29.00	1706	120.74	28.18
207	120.68	29.49	707	122.16	30.29	1207	119.00	29.00	1707	120.76	28.21
208	120.75	29.54	708	122.15	30.30	1208	119.00	29.00	1708	120.78	28.23
209	120.85	29.78	709	122.13	30.30	1209	119.00	29.00	1709	120.70	28.48
210	120.84	29.76	710	122.29	30.29	1210	119.00	29.00	1710	120.71	28.47
211	120.82	29.74	711	122.11	30.31	1211	119.00	29.00	1711	120.72	28.46
212	120.80	29.75	712	122.07	30.30	1212	119.00	29.00	1712	120.73	28.45
213	120.64	29.54	713	122.31	30.25	1213	119.00	30.00	1713	120.76	28.32

214	120.64	29.54	714	122.29	30.25	1214	119.00	30.00	1714	120.75	28.32
215	120.65	29.54	715	122.29	30.26	1215	120.00	29.00	1715	120.77	28.31
216	120.64	29.55	716	122.59	30.44	1216	119.00	30.00	1716	120.78	28.32
217	120.60	29.36	717	122.40	30.27	1217	119.00	29.00	1717	120.56	28.30
218	120.62	29.41	718	122.50	30.74	1218	119.00	29.00	1718	120.56	28.32
219	120.61	29.41	719	122.65	30.72	1219	119.00	29.00	1719	120.56	28.32
220	120.63	29.36	720	122.41	30.43	1220	119.00	29.00	1720	120.23	28.31
221	119.82	28.98	721	122.43	30.43	1221	119.58	30.29	1721	120.95	30.59
222	119.87	28.93	722	121.29	28.20	1222	119.57	30.30	1722	120.90	30.60
223	119.84	28.99	723	121.31	28.18	1223	119.58	30.33	1723	120.92	30.60
224	119.84	28.96	724	121.31	28.19	1224	119.62	30.29	1724	120.95	30.61
225	119.97	28.83	725	121.38	28.25	1225	119.39	30.16	1725	120.93	30.42
226	119.96	28.85	726	121.38	28.27	1226	119.38	30.13	1726	120.93	30.42
227	119.93	28.86	727	121.38	28.26	1227	119.36	30.20	1727	120.91	30.42
228	119.94	28.88	728	121.38	28.25	1228	119.35	30.18	1728	120.90	30.42
229	119.72	28.85	729	121.22	28.19	1229	119.39	30.29	1729	120.80	30.57
230	119.69	28.84	730	121.24	28.20	1230	119.25	30.30	1730	120.83	30.61
231	119.74	28.83	731	121.25	28.19	1231	119.37	30.26	1731	120.88	30.60
232	119.71	28.80	732	121.22	28.19	1232	121.34	29.71	1732	120.89	30.59
233	119.59	28.74	733	121.34	28.30	1233	119.24	30.11	1733	120.82	30.54
234	119.61	28.66	734	121.29	28.26	1234	119.24	30.10	1734	120.87	30.54
235	119.58	28.67	735	121.33	28.30	1235	119.24	30.12	1735	120.86	30.52
236	119.61	28.69	736	121.29	28.28	1236	119.24	30.13	1736	120.85	30.50
237	119.60	28.64	737	121.35	28.17	1237	119.04	30.14	1737	120.82	30.44
238	119.60	28.60	738	121.35	28.16	1238	119.07	30.14	1738	120.82	30.49
239	119.55	28.54	739	121.34	28.17	1239	119.00	30.10	1739	120.82	30.48
240	119.54	28.60	740	121.34	28.17	1240	119.03	30.13	1740	120.82	30.48
241	119.65	29.34	741	121.04	29.25	1241	121.40	29.49	1741	121.12	30.65
242	119.63	29.35	742	121.03	29.25	1242	121.25	29.29	1742	121.08	30.65
243	119.65	29.34	743	121.12	29.20	1243	121.43	29.44	1743	121.10	30.66
244	119.63	29.34	744	121.04	29.22	1244	121.24	29.08	1744	121.10	30.66
245	119.26	29.18	745	121.21	29.04	1245	121.61	29.79	1745	121.21	30.68
246	119.26	29.18	746	121.21	29.06	1246	121.38	29.13	1746	121.23	30.70
247	119.25	29.18	747	121.20	29.05	1247	121.36	29.15	1747	121.25	30.73
248	119.25	29.19	748	121.19	29.06	1248	121.37	29.13	1748	121.25	30.72
249	119.78	29.36	749	121.14	29.11	1249	121.12	29.34	1749	121.20	30.73
250	119.77	29.36	750	121.16	29.11	1250	121.31	29.12	1750	121.19	30.76
251	119.80	29.36	751	121.12	19.11	1251	121.30	29.14	1751	121.23	30.74
252	119.80	29.37	752	121.12	29.11	1252	121.23	29.19	1752	121.12	30.65
253	119.75	29.34	753	120.79	29.10	1253	121.19	29.12	1753	121.10	30.75
254	119.76	29.33	754	120.79	29.13	1254	121.19	29.13	1754	121.12	30.73
255	119.20	29.33	755	120.79	29.10	1255	121.19	29.13	1755	121.13	30.77
256	119.77	29.36	756	120.79	29.10	1256	121.18	29.11	1756	121.16	30.75

257	119.32	29.11	757	120.93	29.24	1257	121.33	29.25	1757	121.08	30.79
258	119.31	29.15	758	120.94	29.24	1258	121.33	29.24	1758	121.07	30.82
259	119.32	29.13	759	120.95	29.23	1259	121.33	29.24	1759	121.05	30.77
260	119.33	29.12	760	120.93	29.23	1260	121.33	29.27	1760	121.10	30.80
261	118.39	29.01	761	120.10	28.67	1261	121.13	30.18	1761	119.73	31.02
262	118.40	29.01	762	120.06	28.64	1262	121.12	30.19	1762	119.73	31.02
263	118.35	29.03	763	120.07	28.66	1263	121.11	30.02	1763	119.78	31.07
264	118.36	29.05	764	120.26	28.18	1264	121.11	30.18	1764	119.84	31.04
265	118.39	29.30	765	120.04	28.70	1265	121.39	30.27	1765	119.89	30.81
266	118.37	29.26	766	120.30	28.67	1266	121.35	30.25	1766	119.91	30.83
267	118.39	29.29	767	120.36	28.54	1267	121.32	30.26	1767	119.92	30.79
268	118.40	29.32	768	120.48	28.69	1268	121.35	30.27	1768	119.95	30.83
269	118.41	29.22	769	120.29	28.59	1269	121.33	30.24	1769	119.92	31.11
270	118.42	29.22	770	120.34	28.61	1270	121.31	30.22	1770	119.94	31.10
271	118.37	29.21	771	120.31	28.57	1271	121.31	30.23	1771	119.57	31.53
272	118.37	29.20	772	120.33	28.61	1272	121.30	30.24	1772	119.73	31.02
273	118.26	29.15	773	120.24	28.82	1273	121.33	30.19	1773	119.73	31.02
274	118.28	29.16	774	120.24	28.16	1274	121.30	30.17	1774	119.67	30.96
275	118.32	29.05	775	120.24	28.82	1275	121.33	30.18	1775	119.73	30.91
276	118.31	29.07	776	120.24	28.82	1276	121.30	30.19	1776	119.71	31.00
277	118.25	29.18	777	120.14	28.71	1277	121.36	30.17	1777	119.89	30.95
278	118.23	29.20	778	120.12	28.73	1278	121.35	30.16	1778	119.85	30.92
279	118.23	29.22	779	120.14	28.71	1279	121.36	30.15	1779	119.88	30.87
280	118.22	29.24	780	120.12	28.69	1280	121.35	30.17	1780	119.85	30.88
281	118.53	28.66	781	119.38	28.52	1281	120.47	28.03	1781	120.65	30.05
282	118.54	28.65	782	119.39	28.50	1282	120.48	28.03	1782	120.64	30.06
283	118.46	28.67	783	119.39	28.51	1283	120.50	28.05	1783	120.64	30.04
284	118.51	28.64	784	119.41	28.49	1284	120.43	28.03	1784	120.69	30.04
285	118.69	28.61	785	119.47	28.47	1285	120.67	27.88	1785	120.73	30.03
286	118.62	28.59	786	119.47	28.48	1286	120.67	27.87	1786	120.73	30.01
287	118.59	28.61	787	119.47	28.48	1287	120.67	27.89	1787	120.68	30.08
288	118.63	28.61	788	119.47	28.49	1288	120.81	27.99	1788	120.68	30.07
289	118.69	28.78	789	119.64	28.35	1289	120.60	27.97	1789	120.69	30.07
290	118.72	28.81	790	119.68	28.34	1290	120.59	27.95	1790	120.69	30.08
291	118.66	28.78	791	119.66	28.35	1291	120.60	27.94	1791	120.65	29.98
292	118.74	28.73	792	119.69	28.35	1292	120.62	27.95	1792	120.65	29.98
293	118.72	28.85	793	119.26	28.36	1293	120.57	27.99	1793	120.69	30.00
294	118.70	28.85	794	119.29	28.35	1294	120.59	28.00	1794	120.17	30.00
295	118.68	28.83	795	119.25	28.37	1295	120.54	28.00	1795	120.72	30.02
296	118.69	28.84	796	119.30	28.35	1296	120.52	28.00	1796	120.71	30.02
297	118.51	28.79	797	119.51	28.36	1297	120.63	27.90	1797	120.71	29.97
298	118.50	28.73	798	119.46	28.32	1298	120.62	27.90	1798	120.74	29.98
299	118.50	28.71	799	119.46	28.31	1299	120.66	27.92	1799	120.72	29.97

300	118.54	28.75	800	119.44	28.32	1300	120.62	27.90	1800	120.73	29.96
301	122.15	30.31	801	119.57	29.83	1301	120.69	28.07	1801	120.79	29.98
302	122.17	30.30	802	119.61	29.81	1302	120.69	28.11	1802	120.78	30.00
303	122.16	30.29	803	119.59	29.82	1303	120.69	28.08	1803	120.47	30.04
304	122.14	30.31	804	119.62	29.80	1304	120.68	28.07	1804	120.79	29.98
305	122.19	30.29	805	119.61	29.70	1305	120.76	28.20	1805	120.97	30.01
306	122.20	30.30	806	119.61	29.70	1306	120.74	28.18	1806	120.93	30.04
307	122.22	30.31	807	119.63	29.72	1307	120.75	28.21	1807	120.95	30.01
308	122.22	30.32	808	119.72	29.66	1308	120.78	28.23	1808	120.98	30.05
309	122.13	30.30	809	119.83	29.76	1309	120.71	28.48	1809	120.53	30.73
310	122.11	30.28	810	119.81	29.74	1310	120.71	28.48	1810	120.54	30.82
311	122.11	30.31	811	119.76	29.80	1311	120.72	28.46	1811	120.55	30.84
312	122.06	30.30	812	119.78	29.81	1312	120.73	28.45	1812	120.52	30.66
313	122.31	30.25	813	119.54	29.90	1313	120.76	28.32	1813	121.00	29.77
314	122.30	30.24	814	119.54	29.90	1314	120.75	28.32	1814	120.94	29.78
315	122.28	30.25	815	119.46	29.84	1315	120.77	28.31	1815	121.03	29.78
316	122.35	30.26	816	119.54	29.90	1316	120.78	28.32	1816	121.02	29.78
317	122.30	30.45	817	119.54	29.90	1317	120.56	28.30	1817	120.78	29.88
318	122.29	30.45	818	119.46	29.94	1318	120.56	28.31	1818	120.78	29.88
319	122.40	30.43	819	119.44	29.95	1319	120.56	28.32	1819	120.79	29.87
320	122.42	30.42	820	119.40	29.91	1320	120.57	28.31	1820	120.80	29.87
321	121.23	28.19	821	119.13	29.81	1321	120.78	30.39	1821	119.92	29.44
322	121.25	28.20	822	119.05	29.86	1322	120.81	30.36	1822	119.95	29.42
323	121.23	28.21	823	119.12	29.89	1323	120.80	30.36	1823	119.96	29.43
324	121.23	28.19	824	119.14	29.87	1324	120.79	30.36	1824	119.92	29.43
325	121.38	28.25	825	118.12	29.07	1325	120.75	30.48	1825	120.08	29.52
326	121.38	28.25	826	118.70	29.40	1326	120.78	30.49	1826	120.05	29.51
327	121.40	28.25	827	118.70	29.40	1327	120.79	30.41	1827	120.04	29.48
328	121.40	28.24	828	118.60	29.40	1328	120.79	30.43	1828	120.02	29.54
329	121.32	28.26	829	118.90	29.85	1329	120.64	30.53	1829	120.02	28.28
330	121.32	28.29	830	118.91	29.84	1330	120.67	30.55	1830	120.02	29.49
331	121.34	28.30	831	118.91	29.83	1331	120.70	30.55	1831	125.02	30.30
332	121.32	28.30	832	118.90	29.82	1332	120.71	30.53	1832	120.01	29.49
333	121.31	28.24	833	118.59	29.51	1333	120.57	30.46	1833	119.61	29.93
334	121.30	28.24	834	118.60	29.48	1334	120.59	30.46	1834	119.15	29.94
335	121.29	28.24	835	118.59	29.47	1335	120.58	30.46	1835	119.15	29.96
336	121.32	28.24	836	118.60	29.45	1336	120.56	30.05	1836	119.90	29.57
337	121.31	28.18	837	118.49	29.42	1337	120.58	30.51	1837	120.01	29.45
338	121.32	28.19	838	118.47	29.47	1338	120.58	30.51	1838	120.01	29.43
339	121.32	28.19	839	118.44	29.46	1339	120.55	30.48	1839	119.97	29.44
340	121.33	28.20	840	118.48	29.43	1340	120.56	30.49	1840	119.98	29.47
341	121.21	29.06	841	121.90	29.15	1341	120.45	30.58	1841	120.19	29.34
342	121.20	29.06	842	121.92	29.16	1342	120.46	30.59	1842	120.16	29.35

343	121.20	29.06	843	121.90	29.15	1343	120.48	30.60	1843	120.16	29.35
344	121.21	29.06	844	121.90	29.16	1344	120.47	30.58	1844	120.18	29.35
345	121.14	29.11	845	121.84	29.47	1345	120.53	30.49	1845	120.06	29.20
346	121.16	29.11	846	121.81	26.48	1346	120.54	30.52	1846	119.99	29.17
347	121.12	29.12	847	121.82	29.48	1347	120.56	30.52	1847	120.02	29.18
348	121.12	29.11	848	121.76	29.48	1348	120.54	30.55	1848	120.25	29.15
349	121.04	29.24	849	121.76	29.29	1349	120.56	30.73	1849	120.14	29.40
350	121.04	29.25	850	121.75	29.35	1350	120.56	30.69	1850	120.12	29.83
351	121.03	29.25	851	121.77	29.38	1351	120.54	30.72	1851	120.18	29.39
352	121.12	29.24	852	121.74	29.40	1352	120.59	30.71	1852	120.17	29.39
353	120.93	29.24	853	121.84	29.21	1353	120.36	30.59	1853	120.11	29.30
354	120.93	29.24	854	121.82	29.22	1354	120.36	30.56	1854	120.23	29.14
355	120.94	29.24	855	121.83	29.21	1355	120.32	30.58	1855	120.43	29.17
356	120.95	29.23	856	121.85	29.20	1356	120.35	30.57	1856	120.12	29.32
357	120.79	29.10	857	121.82	29.12	1357	120.37	30.53	1857	119.94	29.24
358	120.79	29.10	858	121.81	29.12	1358	120.36	30.49	1858	119.96	29.27
359	120.79	29.10	859	121.79	29.14	1359	120.35	30.52	1859	119.98	29.25
360	120.79	29.10	860	121.81	29.13	1360	120.36	30.50	1860	119.96	29.27
361	120.09	28.63	861	121.32	29.98	1361	120.22	30.53	1861	118.71	28.97
362	120.06	28.64	862	121.30	30.00	1362	120.24	30.53	1862	118.69	28.94
363	120.07	28.66	863	121.33	29.98	1363	120.17	30.60	1863	118.70	28.91
364	120.10	28.67	864	121.35	29.98	1364	120.18	30.56	1864	118.73	28.94
365	120.04	28.71	865	121.43	29.86	1365	119.96	30.49	1865	118.65	29.09
366	120.04	28.70	866	121.43	30.00	1366	119.96	30.52	1866	118.65	29.08
367	120.34	28.62	867	121.22	29.99	1367	119.98	30.52	1867	118.64	29.07
368	120.02	28.72	868	121.27	29.99	1368	119.99	30.48	1868	118.63	29.07
369	120.54	28.99	869	121.03	30.09	1369	120.09	30.52	1869	118.41	28.87
370	120.53	28.87	870	121.01	30.07	1370	120.05	30.59	1870	118.40	28.86
371	120.54	28.89	871	120.98	30.08	1371	120.05	30.56	1871	118.38	28.86
372	120.49	28.96	872	121.01	30.10	1372	120.07	30.56	1872	118.37	28.86
373	120.24	28.81	873	121.06	30.16	1373	120.12	30.63	1873	118.45	28.91
374	120.24	28.81	874	121.06	30.14	1374	120.06	30.64	1874	118.46	28.92
375	120.25	28.82	875	121.04	30.18	1375	120.12	30.61	1875	118.42	28.91
376	120.22	28.79	876	121.03	30.21	1376	120.09	30.62	1876	118.44	28.93
377	120.12	28.69	877	121.15	30.14	1377	119.93	30.58	1877	118.60	28.92
378	120.14	28.71	878	121.15	30.02	1378	119.99	30.57	1878	118.59	28.91
379	120.12	28.71	879	121.18	30.16	1379	119.98	30.56	1879	118.64	28.91
380	120.12	28.73	880	121.15	30.14	1380	120.01	30.58	1880	118.65	28.89
381	119.39	28.54	881	120.69	28.07	1381	120.72	29.96	1881	119.20	29.10
382	119.41	28.54	882	120.69	28.11	1382	120.73	29.98	1882	119.20	29.11
383	119.42	28.53	883	120.69	28.08	1383	120.71	29.97	1883	119.22	29.11
384	119.40	28.52	884	120.68	28.69	1384	120.74	29.98	1884	119.24	29.12
385	119.47	28.47	885	120.75	28.19	1385	120.64	30.04	1885	119.17	29.07

386	119.47	28.48	886	120.74	28.18	1386	120.64	30.04	1886	119.13	29.04
387	119.47	28.49	887	120.75	28.21	1387	120.65	30.06	1887	119.12	29.07
388	119.47	28.50	888	120.78	28.23	1388	120.64	30.06	1888	119.09	29.09
389	119.64	28.35	889	120.70	28.48	1389	120.68	30.07	1889	119.30	29.06
390	119.64	28.34	890	120.71	28.47	1390	120.68	30.06	1890	119.26	29.26
391	119.66	28.35	891	120.72	28.46	1391	120.68	30.08	1891	119.27	29.06
392	119.69	28.35	892	120.73	28.45	1392	120.68	30.07	1892	119.28	29.05
393	119.28	28.37	893	120.76	28.32	1393	120.64	29.99	1893	119.19	28.92
394	119.29	28.35	894	120.76	28.32	1394	120.64	29.99	1894	119.17	28.88
395	119.29	28.36	895	120.77	28.31	1395	120.65	29.98	1895	119.18	28.85
396	119.25	28.37	896	120.80	28.32	1396	120.65	29.98	1896	119.16	28.90
397	119.52	28.36	897	120.56	28.31	1397	120.73	30.02	1897	119.13	29.04
398	119.46	28.32	898	120.56	28.31	1398	120.72	30.01	1898	119.09	29.09
399	119.45	28.32	899	120.56	28.32	1399	120.72	30.02	1899	119.12	29.07
400	119.44	28.32	900	120.57	28.31	1400	120.71	30.02	1900	119.17	29.07
401	119.57	29.83	901	120.54	27.51	1401	120.99	30.00	1901	122.82	30.72
402	119.61	29.81	902	120.56	27.52	1402	120.47	29.55	1902	122.83	30.73
403	119.59	29.82	903	120.55	27.52	1403	120.86	30.03	1903	122.82	30.72
404	119.62	29.80	904	120.56	27.52	1404	120.49	29.57	1904	122.80	30.79
405	119.61	29.70	905	120.19	28.08	1405	120.83	29.90	1905	122.08	30.60
406	119.61	29.69	906	123.09	30.24	1406	120.82	29.90	1906	122.09	30.60
407	119.63	29.72	907	120.63	27.46	1407	120.83	29.90	1907	122.08	30.59
408	119.72	29.66	908	130.16	28.15	1408	120.82	29.88	1908	122.08	30.59
409	119.83	29.76	909	125.30	34.65	1409	120.84	30.09	1909	122.78	30.72
410	119.81	29.74	910	120.52	27.75	1410	120.83	30.09	1910	122.79	30.72
411	119.75	29.80	911	125.12	34.55	1411	120.82	30.09	1911	122.79	30.73
412	119.78	29.80	912	125.17	34.70	1412	120.81	30.09	1912	122.79	30.72
413	119.54	29.90	913	127.37	26.30	1413	120.04	29.97	1913	122.57	30.66
414	119.54	29.90	914	124.48	27.58	1414	120.08	29.97	1914	122.57	30.68
415	119.46	29.84	915	120.35	26.20	1415	120.07	29.99	1915	122.56	30.67
416	119.54	29.90	916	120.35	26.20	1416	120.04	29.98	1916	122.56	30.66
417	119.46	29.95	917	120.47	27.26	1417	121.01	29.86	1917	122.51	30.71
418	119.46	29.94	918	120.47	27.26	1418	121.02	29.83	1918	122.51	30.71
419	119.44	29.95	919	120.47	27.26	1419	121.03	29.82	1919	122.53	30.71
420	119.40	29.91	920	120.47	27.26	1420	121.07	29.84	1920	122.53	30.72
421	119.16	29.83	921	120.78	30.39	1421	120.73	29.23	1921	121.62	28.97
422	119.09	29.85	922	120.81	30.36	1422	120.73	29.24	1922	121.63	28.97
423	119.11	29.90	923	120.80	30.36	1423	120.70	29.21	1923	121.61	28.97
424	119.13	29.87	924	120.79	30.36	1424	120.72	29.22	1924	121.58	28.96
425	118.75	29.38	925	120.77	30.46	1425	120.58	29.01	1925	121.52	29.09
426	118.77	29.39	926	120.79	30.44	1426	120.59	29.01	1926	121.55	29.09
427	118.77	29.42	927	120.78	30.41	1427	120.56	29.01	1927	121.57	29.11
428	118.77	29.30	928	120.79	30.43	1428	120.56	29.00	1928	121.60	29.06

429	118.84	29.83	929	120.64	30.53	1429	120.47	28.93	1929	121.40	29.15
430	118.84	29.82	930	120.67	30.55	1430	120.46	28.95	1930	121.43	29.16
431	118.81	29.78	931	120.70	30.55	1431	120.49	28.95	1931	121.42	29.15
432	118.80	29.77	932	120.71	30.53	1432	120.45	28.91	1932	121.42	29.14
433	118.67	29.48	933	120.57	30.46	1433	120.66	29.14	1933	121.38	29.06
434	118.65	29.55	934	120.59	30.46	1434	120.65	29.14	1934	121.37	29.07
435	118.61	29.59	935	120.58	30.46	1435	120.70	29.15	1935	121.36	29.06
436	118.37	29.51	936	120.56	30.05	1436	120.66	29.12	1936	121.35	29.06
437	118.55	29.42	937	120.58	30.51	1437	120.41	28.97	1937	121.29	29.09
438	118.54	29.42	938	120.58	30.51	1438	120.38	28.94	1938	121.31	29.09
439	118.57	29.44	939	120.55	30.48	1439	120.39	28.95	1939	121.31	29.10
440	118.55	29.43	940	120.56	30.49	1440	120.38	28.95	1940	121.31	29.09
441	121.86	29.59	941	120.45	30.53	1441	120.01	28.94	1941	121.21	28.96
442	121.85	29.60	942	120.50	30.56	1442	120.02	28.92	1942	121.13	28.58
443	121.83	29.60	943	120.50	30.57	1443	120.04	28.96	1943	121.13	28.55
444	121.85	29.57	944	120.40	30.54	1444	120.04	28.97	1944	121.12	28.57
445	121.90	29.55	945	120.49	30.69	1445	120.05	28.80	1945	121.07	28.89
446	121.88	29.51	946	120.50	30.70	1446	120.10	28.79	1946	121.06	28.89
447	121.87	29.51	947	120.51	30.71	1447	120.12	28.82	1947	121.01	28.87
448	121.88	29.50	948	120.49	30.70	1448	120.12	28.83	1948	121.01	28.88
449	121.87	29.17	949	120.44	30.63	1449	120.23	29.01	1949	121.06	28.70
450	121.85	29.17	950	120.46	30.62	1450	120.23	29.00	1950	121.10	28.74
451	121.88	29.17	951	120.43	30.62	1451	120.25	29.02	1951	121.11	28.74
452	121.94	29.26	952	120.45	30.61	1452	120.24	29.01	1952	121.10	28.74
453	121.90	29.28	953	120.36	30.63	1453	120.10	28.96	1953	121.30	28.94
454	121.85	29.32	954	120.35	30.64	1454	120.12	28.90	1954	121.32	28.94
455	121.80	29.33	955	120.35	30.65	1455	120.11	28.97	1955	121.30	28.94
456	121.80	29.35	956	120.37	30.64	1456	120.12	28.93	1956	121.26	28.92
457	121.67	29.50	957	120.62	30.58	1457	120.12	29.03	1957	121.10	28.47
458	121.68	29.47	958	120.63	30.57	1458	120.12	29.02	1958	121.10	28.47
459	121.62	29.47	959	120.65	30.56	1459	120.13	29.05	1959	121.10	28.47
460	121.63	29.47	960	120.63	30.55	1460	120.12	29.02	1960	121.10	28.47
461	121.32	30.03	961	119.76	30.78	1461	118.66	28.94	1961	120.00	28.09
462	121.32	30.01	962	119.74	30.77	1462	118.67	28.95	1962	120.01	28.08
463	121.39	30.05	963	119.74	30.76	1463	118.70	28.95	1963	120.04	28.10
464	121.37	29.43	964	119.77	30.74	1464	118.66	28.93	1964	120.03	28.08
465	121.57	30.07	965	119.61	30.57	1465	118.65	29.09	1965	120.26	28.05
466	121.16	29.12	966	119.60	30.57	1466	118.66	29.09	1966	120.23	28.07
467	121.13	29.17	967	119.58	30.55	1467	118.63	29.08	1967	120.27	28.06
468	121.14	28.95	968	119.57	30.53	1468	118.63	29.07	1968	120.27	28.07
469	121.02	30.09	969	119.61	30.76	1469	118.38	28.86	1969	120.23	28.34
470	121.01	30.07	970	119.58	30.78	1470	118.41	28.86	1970	120.22	28.36
471	120.96	30.13	971	119.60	30.78	1471	118.42	28.88	1971	120.23	28.33

472	121.00	30.08	972	119.66	30.77	1472	118.44	28.88	1972	120.23	28.35
473	121.06	30.20	973	119.63	30.52	1473	118.44	28.90	1973	120.20	28.09
474	121.05	30.23	974	119.63	30.53	1474	118.45	28.91	1974	120.19	28.08
475	121.08	30.24	975	119.65	30.54	1475	118.45	28.91	1975	120.19	28.08
476	121.08	30.35	976	119.65	30.55	1476	118.43	28.92	1976	120.19	28.07
477	121.17	30.14	977	119.57	30.67	1477	118.67	28.64	1977	120.14	28.27
478	121.19	30.15	978	119.57	30.65	1478	118.68	28.64	1978	120.11	28.27
479	121.18	30.16	979	119.56	30.68	1479	118.65	28.61	1979	120.10	28.23
480	121.20	30.15	980	119.52	30.70	1480	118.68	28.63	1980	120.12	28.24
481	120.68	28.07	981	120.31	29.40	1481	119.02	29.11	1981	119.50	28.06
482	120.70	28.11	982	120.31	29.41	1482	119.19	29.12	1982	119.49	28.05
483	120.70	28.08	983	120.32	29.42	1483	119.21	29.14	1983	119.50	28.05
484	120.69	28.07	984	120.33	29.43	1484	119.21	29.16	1984	119.51	28.08
485	120.75	28.20	985	120.16	29.83	1485	119.29	29.11	1985	119.67	28.22
486	120.75	28.18	986	120.14	29.81	1486	119.16	29.12	1986	119.67	28.21
487	120.75	28.21	987	120.13	29.78	1487	119.15	29.12	1987	119.78	28.26
488	120.78	28.23	988	120.12	29.81	1488	119.10	29.11	1988	119.67	28.25
489	120.70	28.48	989	120.27	29.82	1489	119.31	29.07	1989	119.57	28.18
490	120.72	28.46	990	120.26	29.83	1490	119.26	29.21	1990	119.55	28.25
491	120.72	28.47	991	120.24	29.81	1491	119.22	29.06	1991	119.52	28.20
492	120.72	28.45	992	120.23	29.81	1492	119.21	29.06	1992	119.54	28.20
493	120.76	28.32	993	120.16	29.53	1493	119.13	29.91	1993	119.65	28.20
494	120.75	28.32	994	120.14	29.57	1494	119.19	29.86	1994	119.45	28.13
495	120.77	28.31	995	120.11	29.55	1495	119.17	29.87	1995	119.42	28.10
496	120.79	28.32	996	120.09	29.51	1496	119.17	28.85	1996	119.45	28.13
497	120.56	28.30	997	120.24	29.71	1497	119.17	29.09	1997	119.59	28.05
498	120.56	28.31	998	120.24	29.71	1498	119.19	29.07	1998	119.59	28.06
499	120.56	28.32	999	120.13	29.71	1499	119.15	29.07	1999	119.57	28.06
500	120.40	28.31	1000	120.17	29.72	1500	119.17	29.06			

References

1. UAEPA. Exposure Factors Handbook (Final Report); US Environmental Protection Agency, Washington: Washington, DC, USA., 2011.

2. MEPC. Technical guidelines for risk assessment of contaminated sites, HJ 25.3-2014; Ministry of Environmental Protection of the People's Republic of China: 2014.

3. XL., D. Exposure factors handbook of Chinese population: adults; China Environmental Science Press: Beijing, 2016.

4. XL., D. Exposure factors handbook of Chinese population: children; China Environmental Science Press: Beijing, 2016.

5. Obiri-Nyarko, F.; Duah, A.A.; Karikari, A.Y.; Agyekum, W.A.; Manu, E.; Tagoe, R. Assessment of heavy metal contamination in soils at the Kpone landfill site, Ghana: Implication for ecological and health risk assessment. Chemosphere 2021, 282, 131007, doi:10.1016/j.chemosphere.2021.131007.

6. USEPA. Supplemental Guidance for Developing Soil Screening Levels for Superfund Sites; US Environmental Protection Agency: Washington, DC, USA., 2002.

7. USEPA. Supplemental Guidance for Developing Soil Screening Levels for Superfund Sites [R]. Office of Solid Waste and Emergency Response, Washington, DC 2001, [OSWER9355.4–24].

8. Cheng, Z.; Chen, L.J.; Li, H.H.; Lin, J.Q.; Yang, Z.B.; Yang, Y.X.; Xu, X.X.; Xian, J.R.; Shao, J.R.; Zhu, X.M. Characteristics and health risk assessment of heavy metals exposure via household dust from urban area in Chengdu, China. Sci Total Environ 2018, 619-620, 621-629, doi:10.1016/j.scitotenv.2017.11.144.

9. Jiang, Y.; Chao, S.; Liu, J.; Yang, Y.; Chen, Y.; Zhang, A.; Cao, H. Source apportionment and health risk assessment of heavy metals in soil for a township in Jiangsu Province, China. Chemosphere 2017, 168, 1658-1668, doi:10.1016/j.chemosphere.2016.11.088.

10. Cao, S.; Duan, X.; Zhao, X.; Wang, B.; Ma, J.; Fan, D.; Sun, C.; He, B.; Wei, F.; Jiang, G. Health risk assessment of various metal(loid)s via multiple exposure pathways on children living near a typical lead-acid battery plant, China. Environ Pollut 2015, 200, 16-23, doi:10.1016/j.envpol.2015.02.010.
